# Sex-based differences in the use of post-fire habitats by invasive cane toads (*Rhinella marina*)

**DOI:** 10.1038/s41598-022-14697-7

**Published:** 2022-06-23

**Authors:** Shannon W. Kaiser, Matthew J. Greenlees, Richard Shine

**Affiliations:** School of Natural Sciences, Macquarie University, Sydney, NSW 2109 Australia

**Keywords:** Ecology, Fire ecology, Invasive species

## Abstract

Wildfires can modify habitat attributes, and those changes may differentially affect males versus females within a species if there is pre-existing niche divergence between the sexes. We used radio-tracking and dissections to study invasive cane toads (*Rhinella marina*), and performed transect counts on native frogs and cane toads 12 months after extensive fires in forests of eastern Australia. Both toads and native frogs were encountered more frequently in burned sites than in unburned sites. Most microhabitat features were similar between burned versus unburned areas, but fire had differential impacts on the ecology of male versus female toads. In burned areas females were less numerous but were larger, in better body condition, and had consumed more prey (especially, coleopterans and myriapods). The impact of fire on attributes of retreat-sites (e.g., temperature, density of vegetation cover) also differed between the sexes. More generally, intraspecific divergence in ecological traits within a species (as a function of body size as well as sex) may translate into substantial divergences in the impacts of habitat change.

## Introduction

In many animal species, males and females differ not only in morphological traits such as body sizes and body shapes, but also in behaviour and ecology^1^. For example, territorial defense by one sex may result in widely different patterns of movement and activity in males and females^2^, or extreme size differences between the sexes (perhaps generated by sexual selection) can modify the kinds of habitat used, and the kinds of prey consumed^3^. In such a species, changes to habitat structure (such as wrought by fire or drought) may impact differentially on the two sexes^4^. That situation makes it impossible to speak in general terms about a species being advantaged or disadvantaged by a specific type of habitat change, because the reality will be more complex: one sex may thrive while the other does not.

Anuran amphibians (frogs and toads) offer excellent model systems to investigate this question, because the sexes frequently differ in body sizes and habitat use. Females typically grow larger than conspecific males^5^, and males tend to stay beside waterbodies to call for mates whereas females move more broadly through the surrounding habitat matrix to feed^6^. We might expect sex-based divergence in body sizes and habitat use to translate into differences in the numbers and types of prey that are encountered, and hence in traits such as feeding rates and prey types^3^. Any change in habitat attributes, such as wrought by wildfire, might differentially affect male and female anurans.

The changing global climate, combined with anthropogenic modifications to vegetation, has resulted in an increased frequency and severity of wildfires in many parts of the world^7^. Fires have many effects, but one important one may be to benefit invasive species rather than native taxa^8^. Many invaders thrive in disturbed habitats, whereas many native taxa do not^9^; and as a result, fire may enable invaders to rapidly expand their range into areas that were previously unsuitable^10^. One invasive species that actively selects disturbed rather than pristine habitats is the cane toad (*Rhinella marina*; formerly *Bufo marinus*), that has been spreading through much of Australia since its introduction to that continent in 1935^11,12^. Given strong sex-based differences in microhabitat use within this species^6^, we predicted that the ecological impacts of intense fires on cane toads would differ between the sexes. For example, waterbody margins (used by breeding males) likely would be less affected by fire than would drier habitats used as foraging areas by female toads^5,6^.

To test that prediction, we studied cane toads in north-eastern New South Wales, in an area subject to massive fires a year earlier. We obtained data by three methods. To quantify abundance of native frogs and cane toads, we conducted nocturnal visual transects (N = 31). To quantify abundance, sizes, condition, and food habits of male and female toads in burned versus unburned areas, we measured 1443 toads that had been collected and humanely euthanized by both volunteer “toad-busters” (N = 1391) and our telemetry study (N = 52), and we dissected 481 of those specimens. To quantify microhabitat use and spatial ecology, we radio-tracked 57 toads to record movement patterns and shelter-site characteristics.

## Methods

### Study species

**Figure 1:**
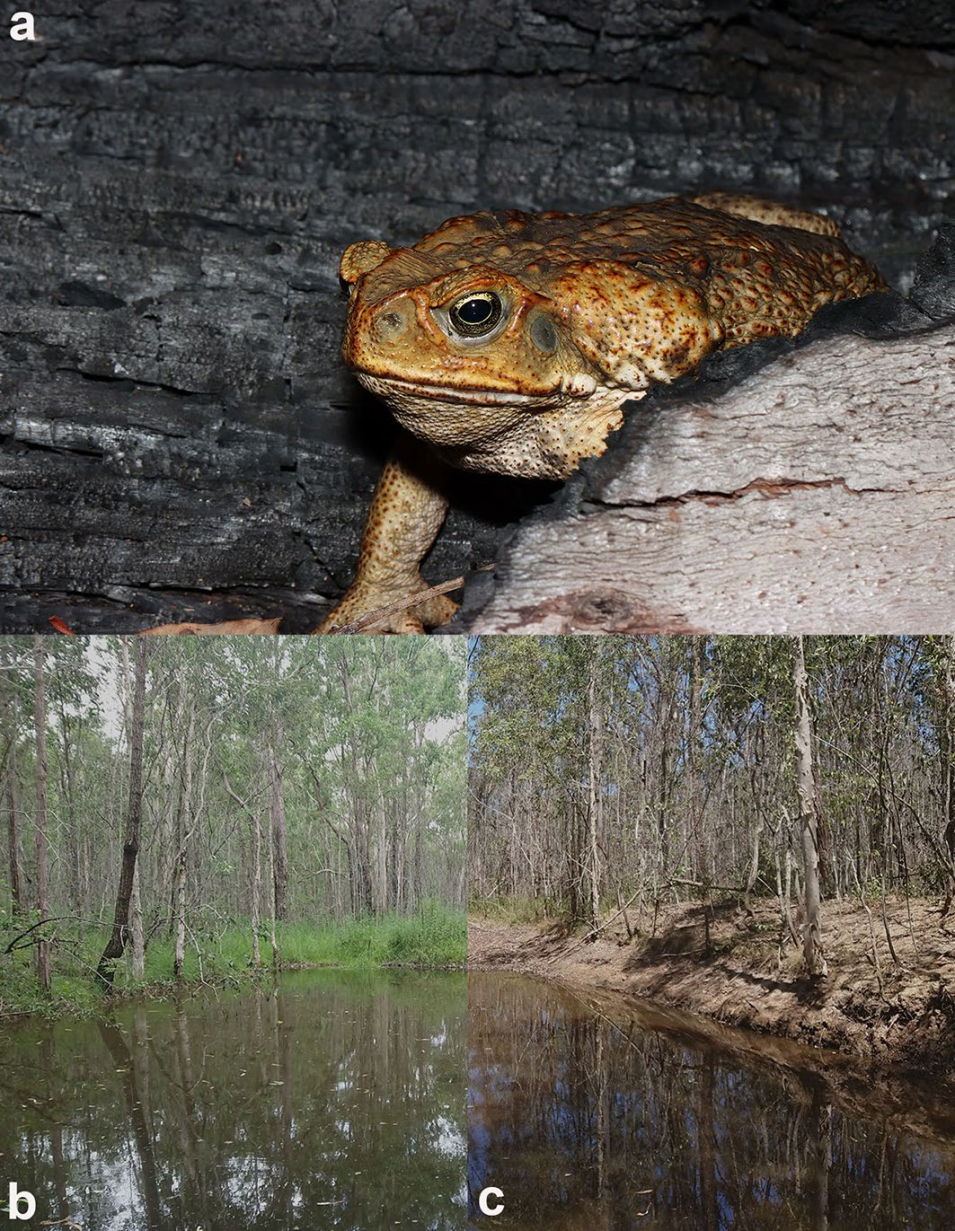
The cane toad *Rhinella marina* (**a**), and unburned, (**b**) and burned (**c**) habitats in which toads were collected and radio-tracked. Photographs were taken from study sites between Casino, Grafton, and surrounds, NSW, by S.W. Kaiser.

Cane toads (*Rhinella marina*) are large (to > 1 kg) bufonids (Fig. 1a). Although native to north-eastern South America, these toads have been translocated to many countries worldwide to control insect pests^12^. Adult cane toads forage at night for insect prey and retreat to moist shelter-sites per day^13^. Small body size (and thus, high desiccation rate) restricts young toads to the margins of natal ponds^14^, but adult toads can survive even in highly arid habitats if they have access to water^13,15^. Cane toads prefer open habitats for foraging^12^, and thus can thrive in post-fire landscapes^16,17^. Cane toads in post-fire landscapes tend to have lower parasite burdens, probably because free-living larvae of their lungworm parasites cannot survive either the fire or the more sun-exposed post-fire landscape^18^.

### Study area

**Figure 2:**
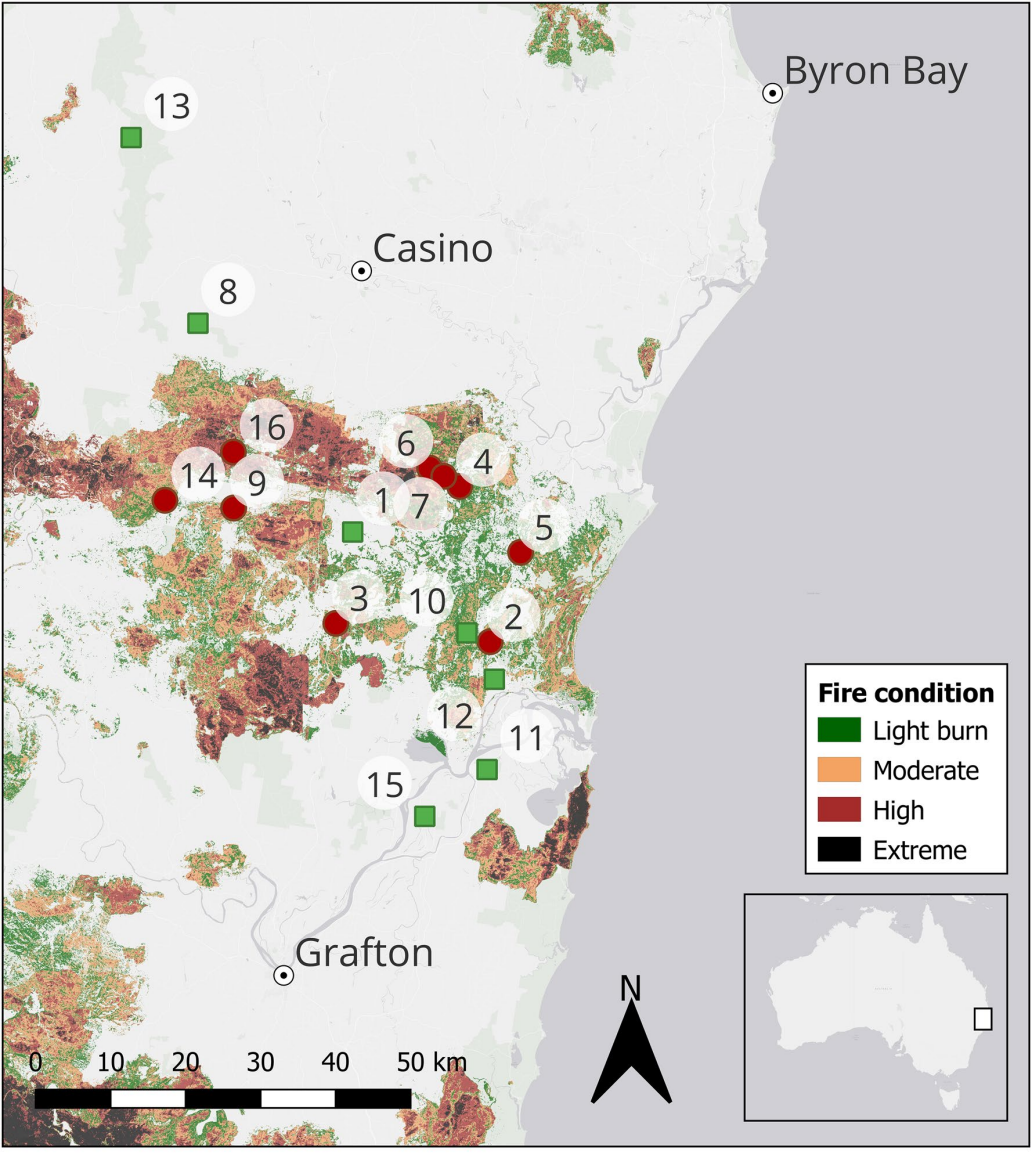
Sampling sites relative to fire history. Sample sites are burned (red circles), and unburned (green squares). See Table 1 for key to sites. The legend shows the extent of burn a year prior to our study. Map created in QGIS 3.22.3. Fire history available from https://datasets.seed.nsw.gov.au/dataset/fire-extent-and-severity-mapping-fesm CC BY 4.0.

East of the Great Dividing Range, near-coastal Clarence Dry Sclerophyll Forests of north-eastern New South Wales (NSW) are dominated by Spotted gum (*Corymbia variegata*) and Pink bloodwood (*Corymbia intermedia*)^19^. Fires are common, but typically cover relatively small areas before they are extinguished. In the summer of 2019–2020, however, prolonged drought followed by an unusually hot summer resulted in massive fires across this region, burning almost 100,000 km^2^ of vegetation^9^. In the current study, the toads we measured and dissected came from several sites within 75 km of the city of Casino (for site locations, see Fig. 2, Table 1, and^18^). The impacts of fire on faunal abundance and attributes shift with time since fire; for example, the abundance of a particular species may be reduced by fire (due to mortality from flames) but then increase as individuals from surrounding areas migrate to the recently-burned site to exploit new ecological opportunities provided by that landscape^8^. We chose to study this system 1-year post-fire, to allow time for such longer-term effects to be manifested.

**Table 1:** Sampling sites and sample sizes for dissected and radio-tracked cane toads (*Rhinella marina*) in New South Wales, Australia.

Site #	Sample site	Latitude	Longitude	Fire treatment	Transects	Telemetry		Dissections	
						Female	Male	Female	Male
1	Bison	− 29.1735	153.03517	Unburned	3	3	2	3	13
2	**Bundjalung**	− 29.3052	153.22500	Burned	9	4	6	8	6
3	*Whiporie Road*	− 29.2832	153.01199	Burned	3				
4	**Bungawalbin**	− 29.1185	153.18288	Burned				7	13
5	*Doubleduke*	− 29.1978	153.26703	Burned	4	4	3		
6	*Million Frog Pond*	− 29.0962	153.14144	Burned	2	5	3		
7	*Neilley's Lagoon*	− 29.1062	153.16096	Burned	3	2	2		
8	Hogarth Range	− 28.9214	152.82159	Unburned				0	20
9	Kippenduff	− 29.1445	152.87088	Burned				36	295
10	**Maclean surrounds**	− 29.2946	153.19296	Unburned	4			39	65
11	*Koala Drive*	− 29.4590	153.22075	Unburned		1	3		
12	*Lewis Lane*	− 29.3505	153.23073	Unburned	3	10	9		
13	Richmond Range NP	− 28.6968	152.72932	Unburned				77	3
14	Smith's and Seery Road	− 29.1352	152.77600	Burned				2	226
15	Woodford Island	− 29.5153	153.13472	Unburned				88	274
16	Wyan	− 29.0770	152.87051	Burned				29	120
	Unspecified			Burned				11	25
	Unspecified			Unburned				16	67
	Total burned			Burned	21	15	14	93	685
	Total unburned			Unburned	10	14	14	223	442
	Grand total				*31*	*29*	*28*	*316*	*1127*

Anuran transects N = 31; radio-tracked toads N = 57; partially dissected toads N = 1443; fully dissected N = 481. Italicized sample sites are subsets of bold sites on lines above; count is independent between sites. Collection localities for dissected toads are aggregated from 87 sites to 10, based on site similarity and distance. Coordinates are averaged to the most central point for each site. Toad locations (Site #) are plotted in Fig. 2.

### Surveys of toad abundance

To quantify toad abundance in burned and unburned sites, one observer (MJG) walked 100-m transects along roads at night (N = 23 and 8 respectively), recording all toads and native frogs (both adult and juvenile). The smaller number of unburned sites reflects the massive spatial scale of the wildfires, which made it difficult to find unburned areas. The transect sites were not the same as those sampled by “toad-busters” (below). We sampled both burned and unburned sites on each night, to de-confound effects of weather conditions with fire treatment. We scored frogs as well as toads to provide an estimate of overall anuran abundance and activity, and so that we could examine toad abundance relative to frog abundance as well as absolute toad numbers.

### “Toad-buster” sample

Because of their ecological impact on native fauna, cane toads are culled by community groups as well as by government authorities^12,20^. We asked “toad-buster” groups to record whether the sites at which they collected toads had been burned during the 2019–2020 fires, or had remained unburned (Table 1). The toads were humanely euthanized (cooled-then-pithed: see^21^). The euthanasia method is brief (a few hours in the refrigerator, followed by pithing) and thus should not have affected any of the traits that we measured. For all of these toads, we measured body length (snout-urostyle length = SUL) and mass, and determined sex based on external morphology (skin colour and rugosity, nuptial pads: see^22^). A subset of toads (chosen to provide relatively equal numbers of males and females, and with equal numbers from burned and unburned sites) was dissected to provide data on mass of internal organs (fat bodies, liver, ovaries), reproductive condition (state of ovarian follicle development) and diet (mass and identity of prey items). To select the subsample of toads for dissection, we took relatively equal numbers of male and female toads from each bag of toads that was provided to us by the “toad-busters”. For logistical reasons, we were unable to dissect all of the toads that had been collected. Overall, we obtained data on morphology, diets and other traits from 481 fully dissected and 1443 partially dissected cane toads.

### Radio-tracking

To explore habitat use and movement patterns, we radio-tracked 57 toads over the course of two fieldtrips (0900–1800 h from 20 Nov 2021 to 6 Dec 2021 and 25 Jan 2022 to 10 Feb 2022). We selected seven sites (4 burned, 3 unburned) within 28 km of Tabbimoble, NSW (see Table 1 for locations and sample sizes of tracked toads). We hand-captured toads found active at night. These were measured, and their sex determined by external morphology (see above) and behaviour (release calls, given only by males: see^23^). We then fitted the toads with radio-transmitters (PD-2; Holohil Systems, Ontario, Canada; weighing ≤ 3.8 g) on cotton waist-belts, and released them at the site of capture. Tracked toads were 88.2–160.9 mm SUL (mass 70.1–546.3 g); thus, transmitters weighed < 10% of body mass (as recommended by^24^). Toads were located daily for the next 5 days using a handheld Yagi directional antenna and a scanning receiver (Australis 26 k; Titley Scientific, Queensland, Australia), and coordinates were recorded using a handheld GPS (Garmin eTrex 10; using UTM). We calculated path straightness as in^24^. We recorded displacement as the distance from the initial refuge; all distances were measured directly in the field rather than estimated from subsequent GIS analyses, except that distance between coordinates was determined in Excel by calculating the hypotenuse between easting and northing measurements over successive days.

We recorded attributes of tracked toad habitat and shelter-sites by visually estimating the percentage cover of environmental variables in a 1-m^2^ quadrat. We estimated vegetation density (understory and canopy) as the percentage of shading over the quadrat at midday. We also estimated mean height of vegetation (understory, canopy, and grass) and the distances from a toad’s diurnal refuge to the nearest road and waterbody. Additionally, we counted the number of vertical stems (5–20 mm thick) and trunks (> 20 mm thick) within the quadrat, and estimated exposure of the toad within its refuge (the percentage of the animal’s body exposed to the naked eye). We then selected a compass bearing at random and walked 20 m in that direction where we rescored all of the above habitat attributes, to quantify habitat features in the broader environment (i.e., not just in microhabitats used by toads). We used those “random” sites to quantify overall habitat attributes of burned and unburned sites. Temperature was recorded by directing a temperature gun (Digitech QM7221) on (or otherwise close-to) toads and at a random point on the ground for random replicates. In total, we gathered radio-tracking data on movements and habitat variables from 57 cane toads, each of which was tracked for 5 days. Recaptured toads were euthanized by cooling-then-pithing.

### Morphological traits

To obtain an index of body condition of toads, we regressed ln mass against ln SUL, and used the residual scores from that general linear regression as our estimate of body condition. Negative residual scores show an individual that weighs less-than-expected based on its body length. Likewise, we regressed mass of the fat bodies, liver and stomach against body mass to obtain indices of energy stores and stomach-content volumes relative to body mass. We scored male secondary sexual characteristics using the system of Bowcock et al.^22^. In their system, three sexually dimorphic traits (nuptial pad size, skin roughness and skin colouration) are scored from 0 to 2, and the scores from those three traits are summed to create a final value (on a 6-point scale) for the degree of elaboration of male-specific secondary sexual characteristics. We scored reproductive condition in adult female toads based on whether or not egg masses were visible during dissection, based on dissected toads from both “toad-buster” and telemetry samples.

### Statistical methods

Data were analysed in R version 4.2.0^25^. We used Linear Mixed Models (LMMs), Generalised Linear Mixed Models (GLMMs) and logistic regressions for our analyses. The R packages ‘tidyverse’^26^, ‘lmerTest’^27^, and ‘performance’^28^ were used.

### Habitat data

We compared habitat variables between burned and unburned sites, and attributes of toads in burned versus unburned sites, using GLMMs (with negative binomial distribution) for count data (models were checked for overdispersion^29^) and LMMs on distance data, using ln-transformations where required to achieve normality. LMMs were used on non-normal percentage data, which were ln- and then logit-transformed (using log[(P + e)/(1 − P + e)], where e is the lowest non-zero number, halved)^30^. We used toad id, site (sampling location) and sampling trip (2019 versus 2020) as random factors.

### Anuran transect data

Counts of toads in burned versus unburned areas were compared both directly via GLMMs with a negative binomial distribution and relative to the numbers of frogs sighted along the same transects (binding the columns in R as ‘number of toads, number of amphibians – number of toads’ and using a GLMM with a binomial distribution). We used site as a random factor.

### Telemetry data

For telemetry data, we analysed response variables via LMMs, and ln-transformed data where relevant to achieve normality.

### Dissection data

We used LMMs for SUL, body mass, body condition and organ mass residuals (e.g., fat body mass relative to body mass). For prey item data, we used a poisson distribution with row number as a random factor, as the negative binomial and beta distribution GLMMs were overdispersed (see^31^). We used LMM for number of prey items and number of prey groups, with site as a random factor. Where models failed to converge, we reduced or removed the error term(s). Analyses were restricted to toads ≥ 70 mm SUL, because animals below this size were difficult to sex. We also performed nominal logistic regression to explore variation in sex ratio and male secondary sexual traits.

### Reproductive condition

We used LMM for male secondary sexual characteristic display, using site as a random factor. For ovary presence, we used a binomial GLMM with a logit link, using site as a random factor. We used a LMM of the residual values from ovary mass relative to body mass (ln-transformed), using site as a random factor.

### Ethics declarations

All procedures were performed in accordance with the relevant guidelines and regulations approved by Macquarie University Animal Ethics Committee (ARA Number: 2019/040-2) and in accordance with ARRIVE guidelines.

## Results

### Effects of fire on habitat attributes

Although the impacts of fire were evident visually in some sites (e.g., Fig. 1b,c), fire had little effect on the microhabitat attributes that we measured in the habitat available to toads (based on random sites; Table 2). Only two variables were significantly affected by fire, with burned habitats containing taller grass and more trunks. The scarcity of statistically significant differences between burned and unburned sites reflects high variation among sites within categories, because mean values for many descriptors differed substantially—for example, unburned sites had higher mean values for canopy thickness, grass cover, and leaf litter (Table 2). In turn, that among-site variation likely was driven by small spatial-scale effects of fire intensity coupled with unmeasured factors such as soil quality and drainage patterns.

**Table 2:** Characteristics of habitats available to cane toads (*Rhinella marina*) in burned versus unburned forests, based on scoring of habitat traits in randomly-selected sites.

	Variable	Burned	Unburned	DF	Effect	P
**Distance/height**						
†	Distance to road (m)	22.01 (1.99)	22.43 (2.17)	1, 7.67	− 0.07	0.95
†	Distance to water (m)	46.74 (2.55)	19.64 (1.61)	1, 7.72	− 1.56	0.16
†	Canopy height (m)	19.76 (0.31)	17.75 (0.35)	1, 8.70	− 2.07	0.07
†	Understory height (m)	2.87 (0.24)	3.10 (0.21)	1, 1.74	1.27	0.35
†	Grass height (cm)	48.03 (2.84)	30.78 (3.24)	1, 7.18	− 2.93	**0.02**
**Percentage cover**						
†	Canopy	6.62 (0.81)	10.43 (1.15)	1, 4.81	1.37	0.23
†	Understory	30.66 (1.32)	27.14 (1.85)	1, 4.67	− 1.64	0.17
†	Bare ground and road	19.34 (1.95)	11.67 (1.86)	1, 4.28	− 0.75	0.49
†	Grass	31.14 (2.29)	40.25 (2.94)	1, 54.15	1.44	0.16
†	Leaf litter	30.84 (2.00)	37.93 (2.68)	1, 7.35	0.29	0.78
†	Mud	2.50 (0.74)	2.21 (0.95)	1, 5.40	− 1.02	0.35
†	Rock	4.93 (1.04)	2.21 (0.92)	1, 3.46	− 1.09	0.35
†	Water	1.47 (0.81)	0.04 (0.04)	1, 4.29	− 1.03	0.36
†	Wood	9.78 (1.17)	5.69 (0.79)	1, 6.68	− 1.91	0.10
**Other**						
§﻿§	Number of stems	2.26 (0.24)	2.86 (0.47)	1, 269	− 0.27	0.79
§﻿§	Number of trunks	0.82 (0.12)	0.32 (0.07)	1, 270	− 3.27	**0.001**
†	Refuge temperature (°C)	35.09 (0.76)	34.87 (0.69)	1, 7.10	− 0.05	0.96

The Table provides information on mean values and standard errors (in parentheses) of habitat variables, and results of statistical tests (Linear Mixed Models and Generalised Linear Mixed Models) comparing burned versus unburned sites. Bold font indicates significant values (P < 0.05).

† Indicates that T and F values were used.

﻿§﻿§ Indicates a negative binomial distribution (Z and χ^2^ values were used).

### Surveys of toad abundance

**Figure 3:**
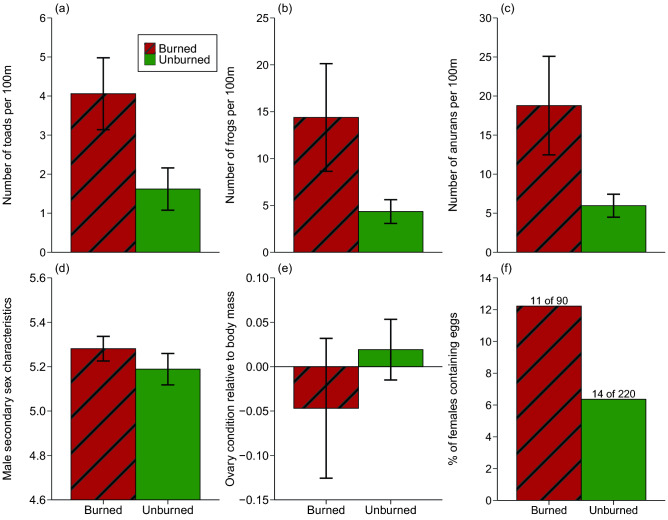
Anuran transect counts per 100 m and reproductive status of dissected cane toads in burned and unburned areas. Panels (**a**–**c**) are from anuran transect data, and show (**a**) the number of toads detected, (**b**) the number of frogs detected, and (**c**) the total number of anurans detected along 100 m transects during nocturnal surveys. Panels (**d**–**f**) are from toads dissected from “toad-buster” and telemetry samples, which show (**d**) the degree of elaboration of secondary sexual characteristics of male cane toads, where skin rugosity, colour and nuptial pad intensity were scored from 0 to 2 and summed to create an index, with higher values representing stronger secondary sexual characteristic intensity; (**e**) ovary condition (ovary mass relative to body mass); and (**f**) percentage of females containing visually-observable egg masses, where text above bars are count and total values. Panels (**a**–**e**) show mean values and associated standard errors.

We obtained data from 31 transect surveys (23 in burned areas, 8 in unburned areas), recording a total of 1197 anurans (306 cane toads, 891 frogs). Compared to unburned sites, our surveys in burned sites revealed more toads (Z = − 2.10, P < 0.04; Fig. 3a) and more native frogs (Z = − 3.13, P < 0.002; Fig. 3b) and thus more anurans in total (Z = − 3.49, P < 0.0005; Fig. 3c). The abundance of toads relative to the abundance of native frogs (primarily green tree frogs *Litoria caerulea*, ornate burrowing frogs *Platyplectrum ornatum*, and striped rocket frogs *Litoria nasuta*) did not differ significantly between burned and unburned areas (Z = − 0.35, P = 0.73) nor did the species richness of native frogs differ between burned versus unburned sites (Z = − 1.19, P = 0.24).

### “Toad-buster” sample

**Figure 4:**
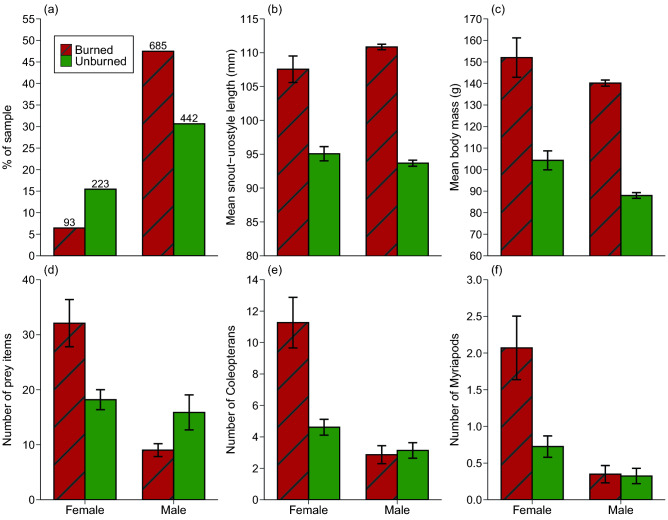
Sample sizes and sex ratio of cane toads (*Rhinella marina*), and number of prey items in the stomachs of dissected toads (from “toad-buster” and radio-tracked toad samples) in burned and unburned areas. Panels show (**a**) the sex ratio for the entire sample (values above bars are counts), (**b**) body length (snout-urostyle length), (**c**) body mass of cane toads, (**d**) the total number of prey items, (**e**) the number of Coleopterans (beetles), primarily scarabs, and (**f**) the number of Myriapods, primarily millipedes. Panel (**a**) shows percentage, panels (**b**–**f**) show mean values and associated standard errors.

We obtained data on external morphology and sex/age class of 1443 cane toads (1127 males, 316 females). Males were more abundant in burned areas than were females, however not significantly so when accounting for pseudoreplication (Z = − 0.083, DF = 1, 1321, P = 0.93; Fig. 4a). We dissected ≥ 481 of these 1443 toads (≥ 231 from burned sites, and ≥ 250 from unburned sites) to obtain data on internal organs and diets. We attempted to select relatively equal numbers of males and females for this more detailed analysis, but the final sample was male-biased because of limited numbers of females from some sites. This analysis used dissected toads from both “toad-buster” and telemetry samples.

### Reproductive biology

Secondary sexual characteristics were well-developed in adult males from both burned and unburned areas, with high scores (5 or above, on a 6-point scale) for 84% of males in burned areas and 80% of males in unburned areas (Fig. 3d). Thus, the overall degree of elaboration of these traits did not differ significantly between males from the two fire-treatment categories (T = 0.55, DF = 1, 7.83, P = 0.59; Fig. 3d). On average, ovaries (relative to body mass) were smaller in female toads from burned areas than from unburned areas, but these differences were not statistically significant (T = 0.36, DF = 1, 5.04, P = 0.74; Fig. 3e). The same was true for the proportion of females containing ovaries with well-developed eggs (Z = − 1.12, DF = 1, 281, P = 0.26; Fig. 3f). This analysis used dissected toads from both “toad-buster” and telemetry samples.

### Toad body size, body condition and mass of fat bodies and stomach contents

Mean body sizes (SULs) were larger for both male and female toads in burned areas (Fig. 4b, Table 3). Mean body mass was affected by a fire*sex interaction, whereby both sexes exhibited higher body mass in burned areas, and with males consistently weighing less than females (Fig. 4c, Table 3).

**Table 3:** Results of statistical analyses (Linear Mixed Models and Generalised Linear Mixed Models) on the effect of fire condition (burned versus unburned) and sex, and their interaction, on the morphology, diet, movements and habitat use of cane toads (*Rhinella marina*).

	Variable	Fire			Sex			Fire*sex		
		DF	Effect	P	DF	Effect	P	DF	Effect	P
**Body size**										
†	Snout-urostyle length	1, 10.78	− 2.95	**0.01**	1, 1316.8	− 0.16	0.88	2, 1305.3	0.72	0.47
†	Body mass	1, 10.93	− 2.71	**0.02**	1, 1316.9	− 4.51	**0.000001**	2, 1305.9	2.60	**0.01**
**Relative to body mass**										
†	Body condition	1, 9.02	− 1.32	0.22	1, 1275.7	4.31	**0.00002**	2, 1182.2	− 1.57	0.12
†	Fat mass	1, 13.15	3.16	**0.01**	1, 443.2	− 1.30	0.19	2, 430.9	− 3.41	**0.001**
†	Stomach mass	1, 8.56	− 0.20	0.84	1, 460.6	− 7.61	**0.000001**	2, 461.8	3.62	**0.0003**
†	Liver mass	1, 9.55	1.20	0.26	1, 459.0	0.98	0.33	2, 460.0	− 1.87	0.06
**Number of prey items**										
†	Total prey	1, 11.16	0.34	0.74	1, 464.4	− 5.11	**0.000001**	2, 462.0	1.71	0.09
†	Total prey groups	1, 12.85	− 0.32	0.76	1, 453.9	− 5.04	**0.000001**	2, 445.6	1.38	0.17
§	Coleopterans		− 0.07	0.95		− 6.12	**0.000001**		2.17	**0.03**
§	Debris		− 1.85	0.06		− 3.79	**0.0001**		2.56	**0.01**
§	Hymenopterans		0.61	0.54		− 2.18	**0.03**		0.59	0.56
§	Larvae		0.36	0.72		− 0.27	0.79		− 0.30	0.76
§	Lepidopterans		− 0.19	0.85		− 3.63	**0.0003**		2.19	**0.03**
§	Myriapods		− 1.34	0.18		− 4.64	**0.000001**		2.63	**0.01**
§	Orthopterans		− 1.68	0.09		− 0.28	0.78		0.28	0.78
§	Other prey		0.30	0.77		− 0.99	0.32		0.48	0.63
§	Spiders		0.99	0.32		− 0.50	0.62		0.07	0.95
**Characteristics of refuges**										
*Distance/height*										
†	Distance to road	1, 13.63	1.39	0.19	1, 53.26	2.20	**0.03**	2, 51.98	− 2.82	**0.01**
†	Distance to water	1, 9.14	− 2.15	0.06	1, 49.10	− 2.26	**0.03**	2, 47.21	1.06	0.30
†	Canopy height	1, 11.98	− 1.75	0.11	1, 49.77	− 2.03	**0.05**	2, 48.51	0.41	0.69
†	Understory height	1, 5.27	0.36	0.73	1, 43.97	0.40	0.69	2, 44.57	− 0.02	0.98
†	Grass height	1, 15.64	− 1.58	0.13	1, 53.30	− 1.75	0.09	2, 52.12	1.99	**0.05**
*Percentage cover*										
†	Canopy	1, 11.62	0.18	0.86	1, 51.28	− 1.30	0.20	2, 53.03	− 1.15	0.26
†	Understory	1, 13.53	− 0.57	0.58	1, 51.76	− 1.29	0.20	2, 51.06	0.90	0.37
†	Bare ground and road	1, 9.92	− 0.42	0.68	1, 51.76	0.49	0.62	2, 49.12	− 1.08	0.29
†	Grass	1, 11.17	− 0.72	0.49	1, 51.58	− 1.61	0.11	2, 49.31	3.35	**0.002**
†	Leaf litter	1, 10.26	0.59	0.57	1, 51.86	− 0.16	0.87	2, 49.38	− 2.13	**0.04**
†	Mud	1, 9.16	0.21	0.84	1, 51.41	− 0.69	0.49	2, 48.37	0.08	0.94
†	Rock	1, 12.58	− 1.16	0.27	1, 53.25	2.49	**0.02**	2, 51.34	− 2.07	**0.04**
†	Water	1, 14.40	1.97	0.07	1, 51.92	− 0.22	0.82	2, 53.20	0.58	0.56
†	Wood	1, 14.47	− 0.03	0.98	1, 53.14	1.18	0.25	2, 52.93	− 2.04	**0.05**
*Other*										
§§	Number of stems		− 1.55	0.12		− 1.74	0.08		1.15	0.25
§§	Number of trunks		− 3.24	**0.001**		− 1.40	0.16		− 0.08	0.94
†	Toad percent visible	1, 15.09	− 0.92	0.37	1, 53.17	− 0.99	0.33	2, 52.21	− 0.20	0.84
†	Refuge temperature	1, 9.26	− 1.17	0.27	1, 51.72	− 0.62	0.54	2, 48.24	2.70	**0.01**
**Telemetry**										
†	Change in shelter sites	1, 13.62	− 0.09	0.93	1, 52.74	− 2.28	**0.03**	2, 52.12	1.16	0.25
†	Distance since previous day	1, 12.37	− 0.90	0.39	1, 51.64	− 2.46	**0.02**	2, 48.90	1.26	0.22
†	Maximum distance in a day	1, 12.89	− 0.25	0.81	1, 52.91	− 1.84	0.07	2, 51.85	1.47	0.15
†	Mean displacement per day	1, 12.54	− 0.55	0.59	1, 52.96	− 1.94	0.06	2, 52.24	1.58	0.12
†	Path straightness	1, 7.70	− 0.73	0.49	1, 28.59	− 0.55	0.59	2, 40.63	0.72	0.47
†	Total displacement	1, 12.47	− 0.59	0.57	1, 52.98	− 1.94	0.06	2, 51.30	1.59	0.12
†	Total distance	1, 12.67	− 0.25	0.81	1, 52.98	− 1.92	0.06	2, 51.66	1.32	0.19

Data on morphology were collected from 481 fully dissected toads and 1443 partially dissected cane toads; data on diet were collected from > 481 dissected cane toads. Radio-tracking data on movements and habitat were collected from 57 cane toads, each of which was tracked for 5 days. Bold font indicates significant values (P < 0.05).

^†^Indicates a normal distribution (Z and χ^2^ values).

^§^Indicates a poisson distribution (T and F values).

^§§^Indicates a negative binomial distribution (Z and χ^2^ values).

Mean body condition (mass relative to length) also differed between the sexes, with males being in better condition (Table 3). Females were heavier-bodied in samples from burned areas than from unburned areas, whereas the reverse was true for males. Our measures of energy stores relative to body size showed that fat bodies were larger in toads from unburned areas (Table 3), whereas relative mass of the liver did not differ significantly between the two habitat types or by sex (Table 3). Mean mass of stomach contents was higher (relative to body mass) in female toads from burned areas than from unburned areas, but higher in males from unburned rather than burned areas (Table 3).

### Feeding rates and prey types

Female toads consumed more prey items than did males in both burned and unburned areas (Table 3, Fig. 4d). The numbers of coleopterans and myriapods consumed were higher for female toads in burned sites than for females in unburned sites, or for males in either type of site (Table 3, Fig. 4e,f); the number of lepidopterans consumed was higher in males in unburned than in burned sites, but no such difference was evident for female toads (Table 3). The number of hymenopterans consumed was higher for female than male toads (Table 3). We did not correct these comparisons for body-mass effects, because there is no clear a priori prediction as to how body size affects prey type.

### Radio-tracking

**Figure 5:**
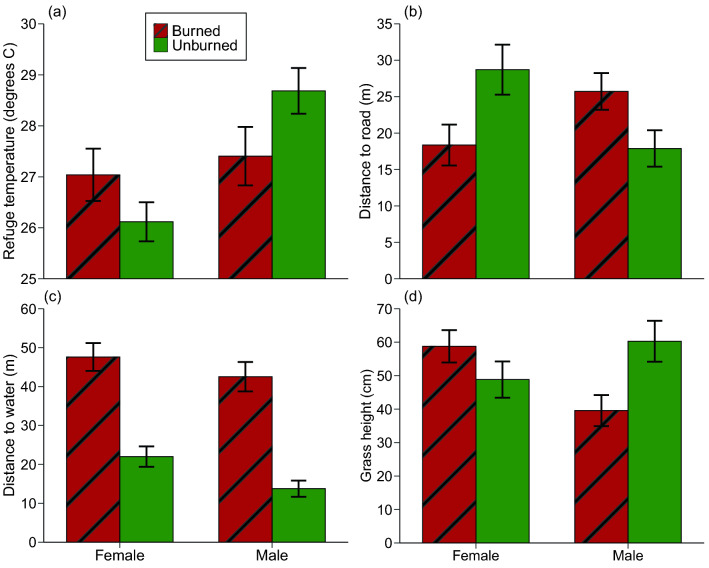
Attributes of refuge-sites (in a 1-m^2^ quadrat) used by radio-tracked cane toads (*Rhinella marina*, N = 57) in burned and unburned areas, as a function of fire condition and toad sex. Panels show (**a**) refuge temperature (measured whenever radio-tracked toads were located), (**b**) distance from refuge to nearest road, (**c**) distance from refuge to nearest waterbody, and (**d**) grass height. Panels show mean values and associated standard errors.

In unburned areas, male toads selected refuge sites with higher mean temperatures than were recorded for burned sites (Table 3, Fig. 5a). That difference reversed in females (Table 3, Fig. 5a). The diurnal shelter-sites of female toads were further from the nearest road in unburned areas than in burned areas, whereas males showed the reverse pattern (Table 3, Fig. 5b). Distance to water was greater in females than in males, and non-significantly higher in burned than in unburned sites (Table 3, Fig. 5c). Female toads selected refuges under taller grass after a site was burned, whereas males did the reverse—that is, they were found under shorter grass in burned sites (Table 3, Fig. 5d).

**Figure 6:**
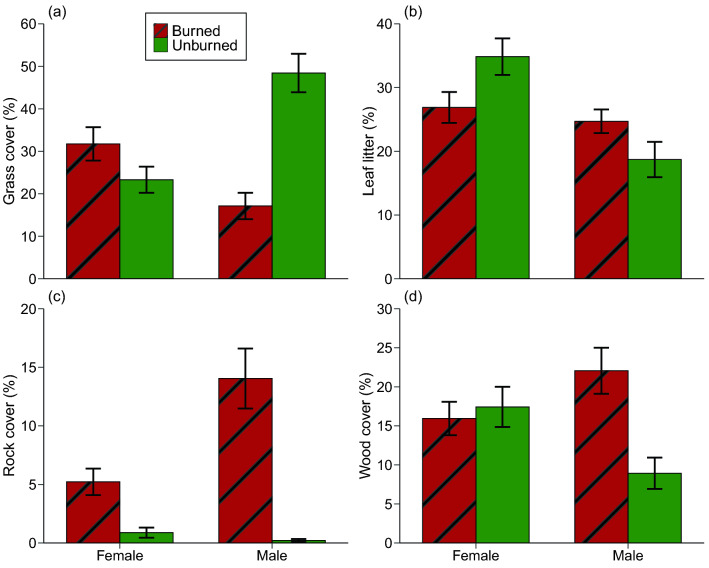
Attributes of refuge-sites (in a 1-m^2^ quadrat) used by radio-tracked cane toads (*Rhinella marina,* N = 57) in burned and unburned areas. Panels show (**a**) percentage of quadrat covered by grass, (**b**) leaf litter, (**c**) rock, and (**d**) wood. Panels show mean values and associated standard errors.

Female toads selected refuges with more grass after a site was burned, whereas males did the reverse (Table 3, Fig. 6a). Male toads selected refuges with greater leaf litter cover in burned sites than in unburned sites, whereas the opposite was true for females (Table 3, Fig. 6b). Toads of both sexes were more likely to use refuge sites containing more rocks in burned sites than in unburned sites (Table 3, Fig. 6c). Males in burned areas were found in refuges surrounded by an abundance of sticks and logs (Table 3, Fig. 6d).

Change in shelter sites were significantly different between sexes (T = – 2.28, DF = 1, 52.12, P = 0.03; Table 3), with radio-tracked females changing shelter sites more frequently (mean proportion ± s.e. = 0.59 ± 0.06) than male toads (0.40 ± 0.06). Daily distances moved were significantly different (T = – 2.46, DF = 1, 51.64, P = 0.02; Table 3), with radio-tracked females moving greater distances (mean ± s.e. = 35.67 ± 5.85 m) than male toads (18.37 ± 4.48 m).

None of the other movement parameters that we measured (maximum distance moved, mean and total displacement, path straightness and total distance moved during the tracking period) were affected by significant fire*sex interactions (Table 3).

## Discussion

Male and female cane toads differ in mean adult body sizes as well as habitat selection^6^—and as a result, may respond differently to perturbations in habitat. Because breeding occurs only in waterbodies, adult male cane toads spend most of their time during the extended reproductive season close to such sites^12^. In contrast, females range widely through the habitat matrix between ponds, and may be better-able to exploit novel opportunities offered by habitat disturbance. For example, well-watered areas around buildings are more likely to be used by female cane toads rather than by males^32^. Nonetheless, some kinds of anthropogenic resource subsidies are equally exploited by male and female cane toads (e.g., beehives^33^).

Bushfire often has negative impacts on native wildlife (e.g., mammals^34^), but can have positive effects on invasive species (e.g., cats, foxes^35^). However, the effects of bushfire on anurans are not well understood, and impacts differ depending on species and life stages. The microhabitats used (arboreal, terrestrial, underground) and associated macrohabitat features (e.g., permanent versus ephemeral streams) will affect both an anuran’s vulnerability to fire and the detectability of the species in surveys such as ours.

After a fire has passed through an area, habitat regrowth may create a new food source for invertebrates, in turn attracting predators including anurans. Consistent with that scenario, we recorded more native frogs and cane toads in burned areas. It is unclear if the anurans that we found in burned areas had survived bushfires, or if they had dispersed post-fire into burned areas. Previous studies suggest that detectability of anurans increases in the months after a fire^36^.

In the present study, we found little effect of intense wildfires on microhabitat parameters in forested areas of north-eastern New South Wales, with the exception of taller grass and more tree trunks in burned areas. That counter-intuitive result reflects the duration of time between the fires and our surveys (12 months), coupled with the fact that the fires were followed by heavy and sustained rainfall, thereby promoting vegetation recovery^37^. Although some parts of the post-fire landscape looked degraded to human eyes (Fig. 1b,c), the characteristics most important to a small moisture-dependent ectotherm (such as the availability of insect prey and moist cool retreat-sites) appear to have been little-affected. Nonetheless, population densities of toads and of native frogs were greater in burned than unburned sites.

The apparently minor effects of fire on habitat attributes masked significant sex-specific impacts of fire on the ecology of cane toads. The toads that were collected in burned areas were larger on average than were those from nearby unburned sites, perhaps reflecting post-fire dispersal into burned habitats by larger toads, and/or faster growth of toads that lived in those sites. It is unclear whether the toads in burned sites were survivors of the fires, having hidden in moist below-ground refuges, or had moved in afterwards. Cane toads are most abundant around human habitation and around waterbodies, two kinds of habitat unlikely to be directly impacted by fire (because of fire-fighting activities and moist soil). A previous study reported that like many other invasive predator species^38^, cane toads are more common in post-fire landscapes^17^.

The shift towards a male-biased sex ratio in post-fire samples (Fig. 4a) may be partly due to a facilitation of collecting around waterbodies, where males are abundant and are easily collected^12^. However, we cannot see any reason why burned areas would differ from unburned areas in this respect; and our telemetry data confirm sex-specific differences in traits such as distances to roads and to water (Fig. 5b,c). Thus, the sex-ratio shift may be real rather than a reflection of sampling bias.

Male cane toads were in lower body condition than female toads, and had consumed fewer prey items. Female toads in burned areas had greater fat stores as well as greater stomach mass. That divergence may reflect spatial heterogeneity in the degree of destruction of vegetation, creating heterogeneity in insect abundance in the post-fire landscape. Wildfires frequently create this kind of spatial variation, with the intensity of flames differing between adjacent sites based on stochastics of wind and temperature, as well as pre-existing habitat differences^39^. The sex that can move freely over broad areas to feed (as female toads did post-fire) can exploit a spatially heterogeneous resource, whereas the sex that is constrained to a more sedentary life around a breeding pond may face a strong tradeoff between feeding and reproducing. In keeping with that idea, male toads in burned areas were more reproductively active (Fig. 3d), and in relatively poor body condition.

Consistent with the idea that the more extensive movements of female toads in burned areas reflected foraging, these animals were in good body condition and contained more prey items than did males in the same sites (Fig. 4d). Interestingly, the sexes differed in the types of prey consumed as well as in rates of foraging. Female toads in burned areas consumed more beetles (coleopterans) and millipedes (myriapods) than did females in unburned areas, or males in either type of habitat (Fig. 4e,f). We have no data on prey availability, and the increased rate of predation on these specific prey types by females might reflect greater abundance and/or greater ease of capture (e.g., due to enhanced visibility of prey items in areas with fewer obstacles on the ground, such as sticks). Burning of vegetation can also release nutrients into the soil, promoting regrowth, and thus attracting a different suite of herbivorous invertebrates and their predators^40^.

Some of the microhabitat-related differences between males and females in our radio-tracking study may reflect broader macrohabitat divergences, notably the proximity of males to waterbodies (Fig. 5c). That pattern might explain why male toads in burned areas took refuge in areas with shorter grass (Fig. 5d), sparser grass, more leaf litter, more rocky ground, and more wood (Fig. 6a-d).

Future research could usefully explore the responses of cane toads to other kinds of habitat modification, and the time course of those responses through time since perturbation. Also, it would be of great interest to look in more detail at the roles of other intraspecifically variable traits—notably body size, but also behavioural syndromes^41^—in determining responses to habitat change. Especially in taxa with multiphasic life histories, and transitions in habitat use with ontogeny as well as a function of sex, the effects of any given environmental change may fall differently on different cohorts. Most obviously, processes that affect the aquatic environment (such as water pollution, perhaps as a direct result of fires) may have far more impact on eggs and larvae than on terrestrial-phase animals; and shifts in the availability of water-sources may be more important to smaller individuals (because of their high rates of desiccation^14^). Thus, habitat changes may be beneficial to some life stages or sexes, while being deleterious to others. Such differential impacts may mediate the overall effect of environmental disturbance on population viability.

## Data Availability

Data on abundance, habitat usage, telemetry and dissections are available from the Dryad Digital Repository at: https://datadryad.org/stash/share/7MkZVodh6ElyVUkdX6jOwY6SlQPBXXFvp09hN-2w1_M.
